# Bimanual coordination and spinal cord neuromodulation: how neural substrates of bimanual movements are altered by transcutaneous spinal cord stimulation

**DOI:** 10.1186/s12984-024-01395-w

**Published:** 2024-06-18

**Authors:** Behdad Parhizi, Trevor S. Barss, Alphonso Martin Dineros, Gokul Sivadasan, Darren Mann, Vivian K. Mushahwar

**Affiliations:** 1 Neuroscience and Mental Health Institute, University of Alberta, Edmonton, AB Canada; 2 Institute for Smart Augmentative and Restorative Technologies and Health Innovation (iSMART), University of Alberta, Edmonton, AB Canada; 3 Division of Physical Medicine and Rehabilitation, Department of Medicine, Katz Group-Rexall Centre for Pharmacy and Health Research, University of Alberta, Edmonton, AB T6G 2E1 Canada; 4 Department of Electrical and Computer Engineering, University of Alberta, Edmonton, AB Canada; 5 Division of Physical Medicine and Rehabilitation, Department of Medicine, University of Alberta, Edmonton, AB Canada

## Abstract

Humans use their arms in complex ways that often demand two-handed coordination. Neurological conditions limit this impressive feature of the human motor system. Understanding how neuromodulatory techniques may alter neural mechanisms of bimanual coordination is a vital step towards designing efficient rehabilitation interventions. By non-invasively activating the spinal cord, transcutaneous spinal cord stimulation (tSCS) promotes recovery of motor function after spinal cord injury. A multitude of research studies have attempted to capture the underlying neural mechanisms of these effects using a variety of electrophysiological tools, but the influence of tSCS on cortical rhythms recorded via electroencephalography remains poorly understood, especially during bimanual actions. We recruited 12 neurologically intact participants to investigate the effect of cervical tSCS on sensorimotor cortical oscillations. We examined changes in the movement kinematics during the application of tSCS as well as the cortical activation level and interhemispheric connectivity during the execution of unimanual and bimanual arm reaching movements that represent activities of daily life. Behavioral assessment of the movements showed improvement of movement time and error during a bimanual common-goal movement when tSCS was delivered, but no difference was found in the performance of unimanual and bimanual dual-goal movements with the application of tSCS. In the alpha band, spectral power was modulated with tSCS in the direction of synchronization in the primary motor cortex during unimanual and bimanual dual-goal movements and in the somatosensory cortex during unimanual movements. In the beta band, tSCS significantly increased spectral power in the primary motor and somatosensory cortices during the performance of bimanual common-goal and unimanual movements. A significant increase in interhemispheric connectivity in the primary motor cortex in the alpha band was only observed during unimanual tasks in the presence of tSCS. Our observations provide, for the first time, information regarding the supra-spinal effects of tSCS as a neuromodulatory technique applied to the spinal cord during the execution of bi- and unimanual arm movements. They also corroborate the suppressive effect of tSCS at the cortical level reported in previous studies. These findings may guide the design of improved rehabilitation interventions using tSCS for the recovery of upper-limb function in the future.

## Introduction

One impressive capability of the human motor system is coordinated movement of the hands to accomplish a task. A large variety of functional activities require some degree of coordination and collaboration between the two hands. Yet, many of these daily activities are impaired by neurological conditions such as spinal cord injury (SCI). For instance, cervical SCI results in prolonged movement time and lower peak velocity in a unimanual reach-to-grasp task compared to non-injured individuals. The same kinematic measures are exacerbated during a bimanual version of the reach-to-grasp task [5]. Restoration of the ability to use the hands in a coordinated manner can substantially improve independence in the performance of daily activities after SCI.

Transcutaneous spinal cord stimulation (tSCS) has emerged as a non-invasive neuromodulatory technique that has the potential to reverse sensory and motor loss after SCI [15, 16], U. S. [21–23, 40]. Cervical tSCS promoted both immediate and long-term improvement in hand and arm function [14, 23]. However, motor gains in the upper limbs after tSCS are commonly reported by clinical tools that evaluate each limb’s function in isolation of the other using unimanual tasks [23]. Moreover, the underlying mechanisms of cervical tSCS are investigated using static tasks that only record muscle responses unilaterally [34, 43]. While this technique has produced promising results, and may become a versatile clinical tool, the utility of tSCS as a means for improving bimanual motor performance is not yet known. To address the therapeutic effect of tSCS on bimanual actions, it is necessary to first identify how tSCS modulates bimanual motor performance and the activity of brain areas subserving it.

To date, a number of studies have investigated the underlying neural substrates of tSCS along the spinal neuroaxis [1, 8, 34, 35, 43]. Both computer modelling and electrophysiological studies have provided substantial evidence that tSCS primarily recruits large-to-medium afferent fibers in the posterior root and dorsal horn of various spinal segments [8]. Moreover, a combination of neural structures is activated with increasing stimulation intensity, including group Ib afferents, group II muscle spindle afferents, and spinal interneurons [1, 15]. Importantly, these effects are not restricted to the site of stimulation, and propagate to remote segments of the spinal cord [37]. We explored the spinal multi-segmental effect of tSCS in two recent studies in neurologically intact individuals. We demonstrated that cervical tSCS suppresses the H-reflex in the soleus muscle of the leg while lumbar tSCS facilitates H-reflex in the flexor carpi radialis (FCR) muscle of the arm [1, 37].

While numerous other studies have investigated the mechanisms by which tSCS recruits neural structures at the spinal cord level, only a few studies alluded to the cortical effects of tSCS. Benavides et al. reported that cervical tSCS with 5 kHz carrier frequency does not modulate the amplitude of motor evoked potentials (MEPs) in proximal and distal arm muscles [3]. In the absence of the carrier frequency, the MEP amplitude increased, suggesting a cortical inhibitory effect when the tSCS waveform is modulated with 5 kHz carrier frequency. Similarly, in our own work, we found that the amplitude of MEPs in the FCR muscle remain unchanged during tSCS when applied with a 10 kHz carrier frequency over the C3–4 and C6–7 spinous processes [37]. Although the majority of studies use MEPs as a measure of cortical excitability, further knowledge can be captured by cortical oscillations recorded by electroencephalography (EEG). Both measures reflect motor cortical excitability, but the excitability is likely driven by different neural processes. Thus, cortical oscillations are an alternative approach to understanding the physiological effects of tSCS, especially that cortical regions (along with their activation patterns) are prominent to explore the processes underlying bimanual movements. A recent study reported that tSCS did not have a consistent effect on sensorimotor cortical oscillations among study participants, but those who received the highest intensities of stimulation showed cortical inhibition [32].

Most research studies aiming to investigate the effects of tSCS have focused on conditions where the upper limbs are static (either at rest or exerting some levels of isometric muscle contraction), and responses are recorded unilaterally by utilizing measures such as MEPs and H-reflexes [3, 43]. However, the question of how tSCS induces modulation of sensorimotor cortical oscillations during bimanual movements is yet to be addressed. In the present study, we assessed the effect of cervical tSCS on sensorimotor cortical regions using EEG during the execution of dynamic unimanual and bimanual movements. Two scenarios were expected in the presence of tSCS: cortical excitation as a result of an increase in sensory afferent volleys, or cortical inhibition as a result of inhibition of nociceptive input and as a consequence of the presence of a carrier frequency in the tSCS waveform [3, 13, 24, 46]. We hypothesized that (1) the kinematics of the reaching movements are improved when cervical tSCS is administered; (2) tSCS induces event-related synchronization (ERS) in cortical oscillations indicative of cortical inhibition; and (3) tSCS elevates the level of interhemispheric connectivity during the execution of movements.

To address these hypotheses, we recruited neurologically intact (NI) participants to perform three types of goal-directed arm reaching movements using a KINARM exoskeleton facilitated by a virtual reality (VR) display. These movements were: (1) unimanual visually-guided reaching (VGR); (2) dual-goal bimanual VGR; and (3) common-goal bimanual VGR. The same three tasks were repeated in the presence of cervical tSCS applied over the C3–4 and C6–7 (cervical) spinous processes. Movement kinematics were tested by measuring reaction time (RT), movement time (MT) and movement error during the execution of the movements. Cortical activity was recorded via EEG electrodes while the participants performed the reaching movements. Alpha (8–12 Hz) and beta (13–30 Hz) band cortical activity associated with sensorimotor processes was computed using spectral power. Interhemispheric connectivity between the right and left primary motor (M1) and somatosensory (S1) cortices were also evaluated.

## Materials and methods

### Participants

Twelve (12) participants were recruited aged 19 to 36 years. All participants were right-handed on the basis of self-report, had normal or corrected vision through the use of contact lenses or glasses, and had no history of neurological conditions.

### Experimental design

**Fig. 1:**
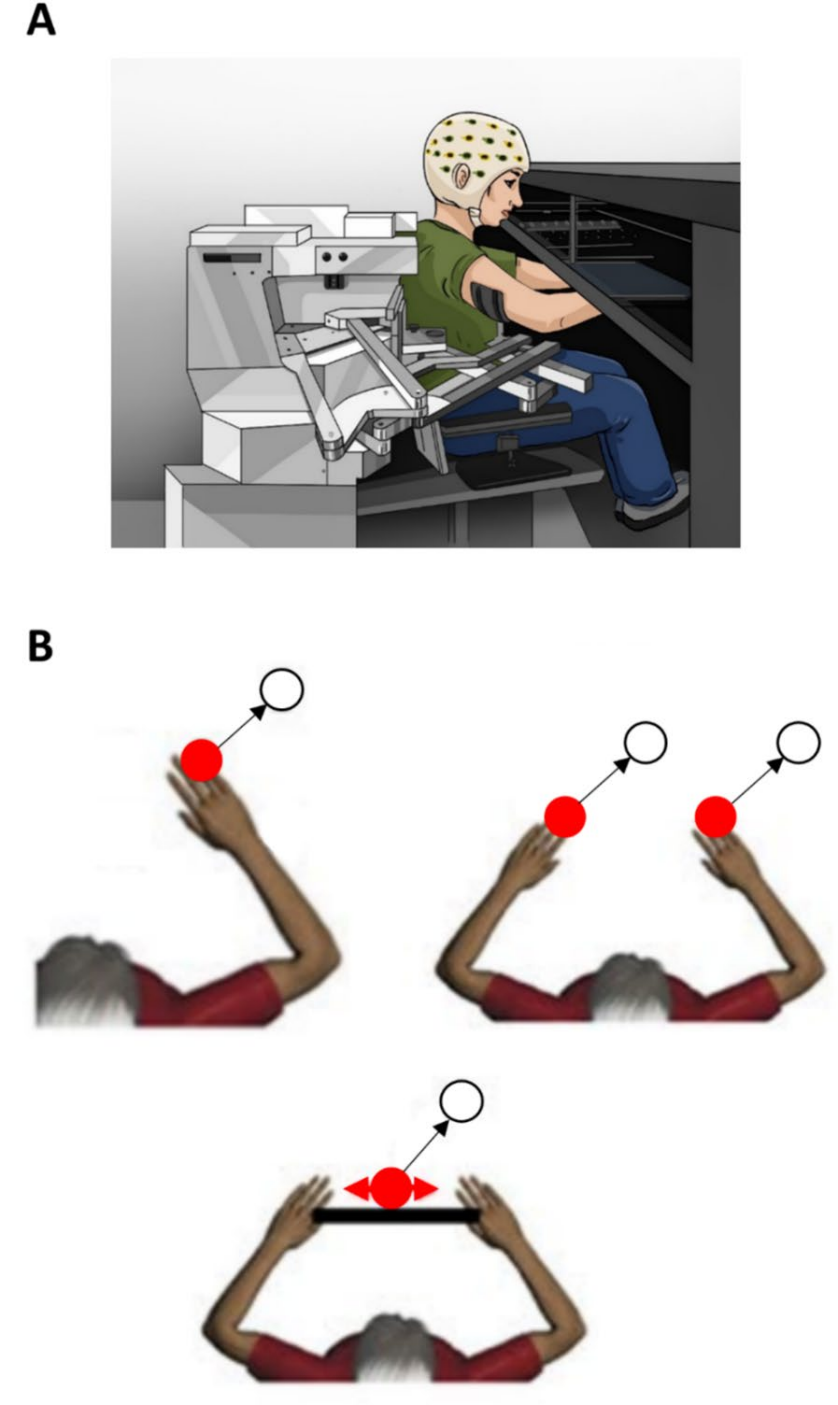
Experimental design. **A** Illustration of the KINARM exoskeleton robotic platform and experimental setup. Participants performed visually-guided reaching tasks guided by a virtual reality display. **B** Representation of the visually-guided tasks: top left—unimanual movement, top right—bimanual dual-goal movement, and bottom—bimanual common-goal movement. All the movements started from a home position (red circle) and ended on a target (white circle)

Participants were seated in front of a KINARM exoskeleton to perform the VGR movements (BKIN Technologies Ltd, Kingston, ON, Canada) (Fig. 1A). The KINARM exoskeleton allows the performance of movements around the elbow and shoulder joints in the horizontal plane. The participants were able to interact with a virtual reality screen that projected the task environment, and the participants’ limbs were supported against gravity by the exoskeleton. The segments of the KINARM were adjusted to accommodate each participants’ limb geometry, and the arm, forearm and hand were supported by troughs attached to adjustable 4-bar linkages. Participants received no assistance from the robot while completing the experimental tasks. The selection of the KINARM as our primary research tool was driven by several considerations. First, the KINARM allows for the design of both custom and standardized tasks tailored for sensorimotor investigations, ensuring the use of an approach that is specific to the function under scrutiny. A salient feature of the KINARM is the guaranteed consistency in task delivery and execution across participants. Such uniformity is pivotal as every participant is exposed to an identical testing paradigm. Second, the KINARM's bilateral design enhances its versatility, accommodating both unimanual and bimanual motor tasks, thus broadening the scope of possible experimental investigations. Lastly, the KINARM can be adjusted to accommodate the physical variations of different participants.

Participants performed three movements facilitated by the exoskeleton (Fig. 1B): (1) unimanual VGR where they were instructed to reach with their right arm to a virtual peripheral target on the top right corner from a home position; (2) bimanual dual-goal VGR where each arm separately but simultaneously performed home-to-target reaching movements to two peripheral targets on the top-right corner of the home positions; and (3) bimanual common-goal VGR where participants moved a ball on top of a horizontal bar connecting both their hands to a peripheral target through cooperative movement of the two arms, each holding one end of the bar. The ball could roll to the sides of the bar if the orientation of the bar deviated from horizontal, thus participants were instructed to maintain the horizontal orientation of the bar throughout the movement. The movement was rejected if the ball fell off of the bar*.* Each reaching task was performed twice, once with and once without stimulation. Therefore, a total of 6 tasks were performed by each participant. The tasks were randomly presented to the participants using simple randomization, and each task was repeated 20 times. Study participants were instructed to move as quickly and as accurately as possible from the home position to the final target. During the KINARM calibration and adjustment steps, participants were exposed to one trial of each task.

Participants started each movement from a home position where their index finger was aligned with a 1.0 cm radius circle and moved to a peripheral target of the same size positioned on the upper right corner (10.0 cm to the right and 10 cm to the top) extending their elbow. Before the movement began, participants held the tip of their index finger on the home position for 750 ms while it was colored red. The color of the home position turned green as the go signal, and the peripheral target appeared on the VR display in red. Once the participant reached the target, they were required to hold their index finger for another 750 ms until the target turned green. At this moment, the home position reappeared and the participant returned back to the home position and waited for 1750 ms for the next repetition of the movement to start. In the bimanual dual-goal task, the same procedure was applied except that each arm performed the reaching movement to two separate targets simultaneously. In the case of the bimanual common-goal task, the movement started from a home position between the two hands located in the middle of the horizontal bar. Participants moved from the home position to the target position on the upper right corner. To ensure similar voluntary cortical drive across all experimental tasks, all movements were performed against a load equivalent to 5–10% of the tricep brachii (TB) maximum voluntary contraction (MVC). To obtain MVC, participants performed three trials of isometric maximal voluntary elbow extension. The KINARM exoskeleton was then programmed to produce a force in the direction opposite to the movement direction and equivalent to 5–10% elbow extension MVC. To compare the effects of cervical tSCS on sensorimotor cortical oscillations and movement kinematics, all tasks were repeated twice, once with and once without tSCS.

### Transcutaneous spinal cord stimulation

**Fig. 2:**
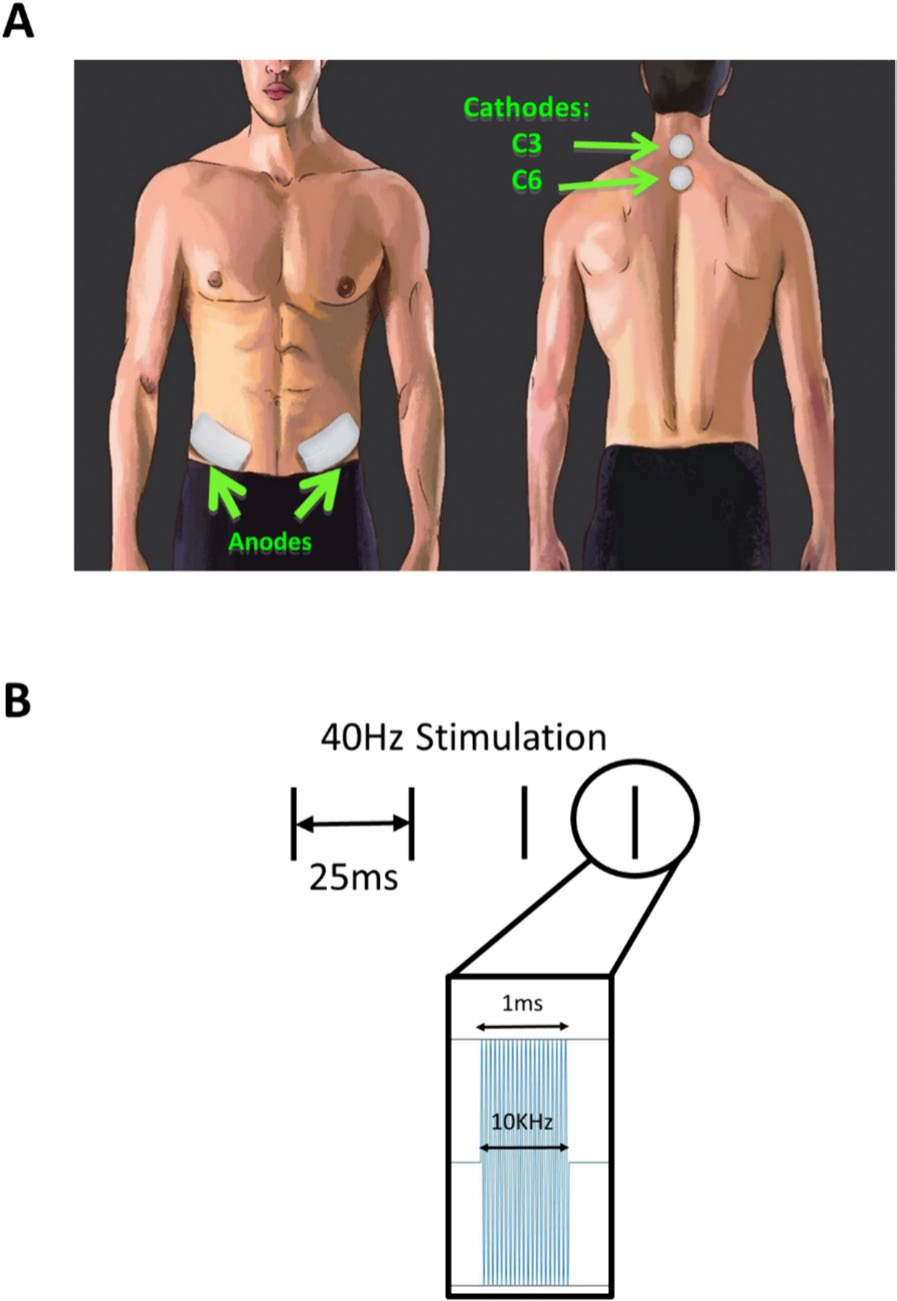
Transcutaneous spinal cord stimulation. **A** Cervical tSCS were delivered through cathodic electrodes placed midline at C3–4 and C5–6 spinous processes. Two anodic electrodes were placed bilaterally over the iliac crests. **B** Stimulation waveform: 1ms long pulses with a carrier frequency of 10 kHz are delivered at 40 Hz

Biphasic cervical tSCS was delivered by two constant current stimulators each having one output channel (DS8R, Digitimer, Hertfordshire, UK). Two cathode electrodes, 3.2 cm diameter (Axelgaard Manufacturing Co., Ltd., United States), were placed midline at C3–4 and C6–7 spinous processes, and two 5 × 7 cm rectangular electrodes were placed bilaterally over the iliac crest as anodes (Fig. 2A). We used a modulated waveform [2] consisting of bursts of ten 100µs-long biphasic square pulses (enveloped in a 1ms pulse) repeated at a frequency of 40 Hz (Fig. 2B).

We used spinal-evoked potentials to determine the stimulation intensity [3]. Single 1ms-long biphasic pulses without the 10 kHz carrier frequency were delivered to both cathodes simultaneously and the stimulation intensity was defined as the minimum amplitude required to evoke potentials in electromyographic (EMG) recordings from the bicep brachii (BB) muscle that were 50 µV peak-to-peak amplitude above background muscle activity in 5 out of 10 trials. This intensity was then used for the continuous stimulation that included the 10kHz carrier frequency. Participants reported a strong buzzing or vibration-like sensation at the cathodic sites as well as tolerable discomfort associated with neck muscle contraction and/or skin irritation. Stimulation was turned on a few seconds prior to the initiation of a movement task and turned off immediately after the completion of 20 repetitions of that task. Therefore, for each task, tSCS remained on for about 3–4 min including the time prior to the initiation of data collection for each movement task.

### Quantification of movement kinematics

RT and MT were calculated using a method introduced by Coderre and colleagues [6] that is based on identification of movement onset and offset. Accordingly, RT was defined as the time interval between the appearance of the peripheral target and the onset of movement. MT was the time interval between movement onset and offset. We also measured movement root mean square error (RMSE) to evaluate the straightness of the participants’ movement. Ideally, each reaching movement should be on the straight line between the home position and peripheral target. RMSE measured the deviation between the real hand coordinates and the closest point (perpendicular distance) to the ideal line.

### Electroencephalography

All EEG recordings were obtained using a 64 channels Brain Vision Recorder (Brain Products, Gilching, Germany) according to the international 10–20 system [27]. Data were recorded and sampled at 1000 Hz. During EEG recordings, the AFz and TP10 electrodes were used as ground and reference, respectively. All data were then re-referenced to the average of electrodes TP9 and TP10 during offline processing. The impedance of each electrode was kept below 5 kΩ and was repeatedly checked throughout the experiment. Since EEG data are prone to unwanted electrophysiological noise, we instructed the participants to sit still and minimize eye blinks and neck movement to ensure high quality recordings.

EEG data were pre-processed using a Butterworth band-pass filter between 0.1 and 200 Hz and notch filtered at 60 Hz. One complication in EEG recordings with nearby surface stimulation is the high-amplitude artifacts associated with stimulation. A recent study showed that EEG recordings are feasible in the presence of tSCS and artifacts were only manifested at the frequency of stimulation in the spectral density analysis [33]; therefore, tSCS posed no detrimental effects on the EEG frequency domain analyses in this study. Given that the artifact occurred every 25ms (stimulation at 40 Hz), no artifact removal techniques were used, such as artifact subspace reconstruction (ASR), to avoid discarding meaningful EEG information. This approach was successfully used in a previous study [32].

### Computing spectral power and coherence

Since the data were continuously recorded during each task, we first split the data into 20 repetitions and then concatenated them. The concatenated data were then used to compute spectral power and coherence between EEG channels for each task. Spectral power was calculated over 1024-point FFT segments with zero over-lap using the following formula [25]:

$${P}_{x}\left(f\right)= \frac{1}{n} \sum_{i=1}^{n}{C}_{i}\left(f\right)* {C}_{i}^{*}(f)$$

where ${P}_{x}\left(f\right)$ is the spectral power for EEG channel x and ${C}_{i}\left(f\right)$ is the fourier transform of data segment i of EEG channel x. Alpha (8–12 Hz) and beta (13–30 Hz) band information for the electrodes over the left M1 (C3 electrode) and S1 (CP3) were included in this study, and the average of power within each band was calculated.

Coherence was then calculated with the following formula [19]:

$${Coh}_{xy}\left(f\right)= \frac{{\left|{P}_{xy}(f)\right|}^{2}}{{P}_{xx}\left(f\right)* {P}_{yy}(f)}$$

where ${P}_{xy}\left(f\right)$ is the cross spectral power of EEG signal x and signal y, and ${P}_{xx}\left(f\right)$ and ${P}_{yy}(f)$ are the spectral power of EEG signal x and y, respectively. Coherence is a scalar value ranging between 0 and 1 and describes the strength of coupling between two signals. Confidence interval at α = 0.95 quantile of the coherence was measured by cl = 1–(1−α)^1/(L−1)^ where L is the number of segments [41]. Right and left interhemispheric coherence was computed for M1 (C3–C4 electrodes) and S1 (CP3–CP4 electrodes) cortical regions in the alpha and beta bands. Only coherence values above the cl threshold were accepted.

### Stimulation artifact removal

It was previously suggested that the effect of stimulation on frequency domain analyses is contained at the stimulation frequency, manifested as an obvious transient high-amplitude peak in the spectral power of the EEG signal [33]. As suggested by [33], notch filtering in the frequency domain and superposition of moving averages in the time domain are the optimal approaches to eliminate the contamination of the frequency bands of interest such as alpha and beta caused by the stimulation artifact. Here, we introduce an alternative approach to remove the stimulation artifact from the EEG time series data and as a consequence, reduce the possible detrimental effect of stimulation artifacts on the spectral power especially those spreading into the alpha and beta bands.

In this study, stimulation frequency was set at 40 Hz and therefore stimulation artifacts should ideally be seen in the EEG time series every 25 ms, each with a duration of 1ms. However, the stimulation artifacts captured by the EEG data persist for 7-11ms. Our approach to remove these artifacts consisted of multiple steps: (1) the time series data were inspected to find the high amplitude peaks produced by the stimulation artifacts; (2) the first and the last point of the stimulation artifact waveform spanning around the peak point in step 1 were found (stimulation artifacts varied slightly in duration for different participants); (3) the average of the EEG data between two consecutive stimulation artifacts (i.e., the last data point of artifact i and the first point of artifact i + 1) was calculated; and (4) the stimulation artifact (i.e., artifact i) waveform (from the first to the last data point detected in step 2) was replaced by the average value calculated in step 3. Through this approach, the stimulation artifact data points were effectively replaced by the average value of the succeeding EEG data points; therefore, suppressing the negative contribution of stimulation artifacts to the spectral power of the nearby bands.

### Statistical analysis

We used a paired samples T-Test to compare the means of RT, MT, movement error, spectral power, and coherence between tasks without and with cervical tSCS (No-tSCS and tSCS) for each movement type. This statistical design allowed us to compare the effect tSCS on each task solely. Descriptive statistics are shown as mean ± standard error, unless otherwise stated. Statistical significance was set for p ≤ 0.05. All statistical analyses were performed with SPSS Statistics (IBM, Chicago, IL, United States).

## Results

Across all participants, cervical tSCS intensity was 38.66 ± 10.66 mA during all experimental tasks (range: 23–61 mA; median: 36.5 mA). The maximum stimulation amplitude of 61 mA was used in only one participant.

### Movement kinematics

**Fig. 3:**
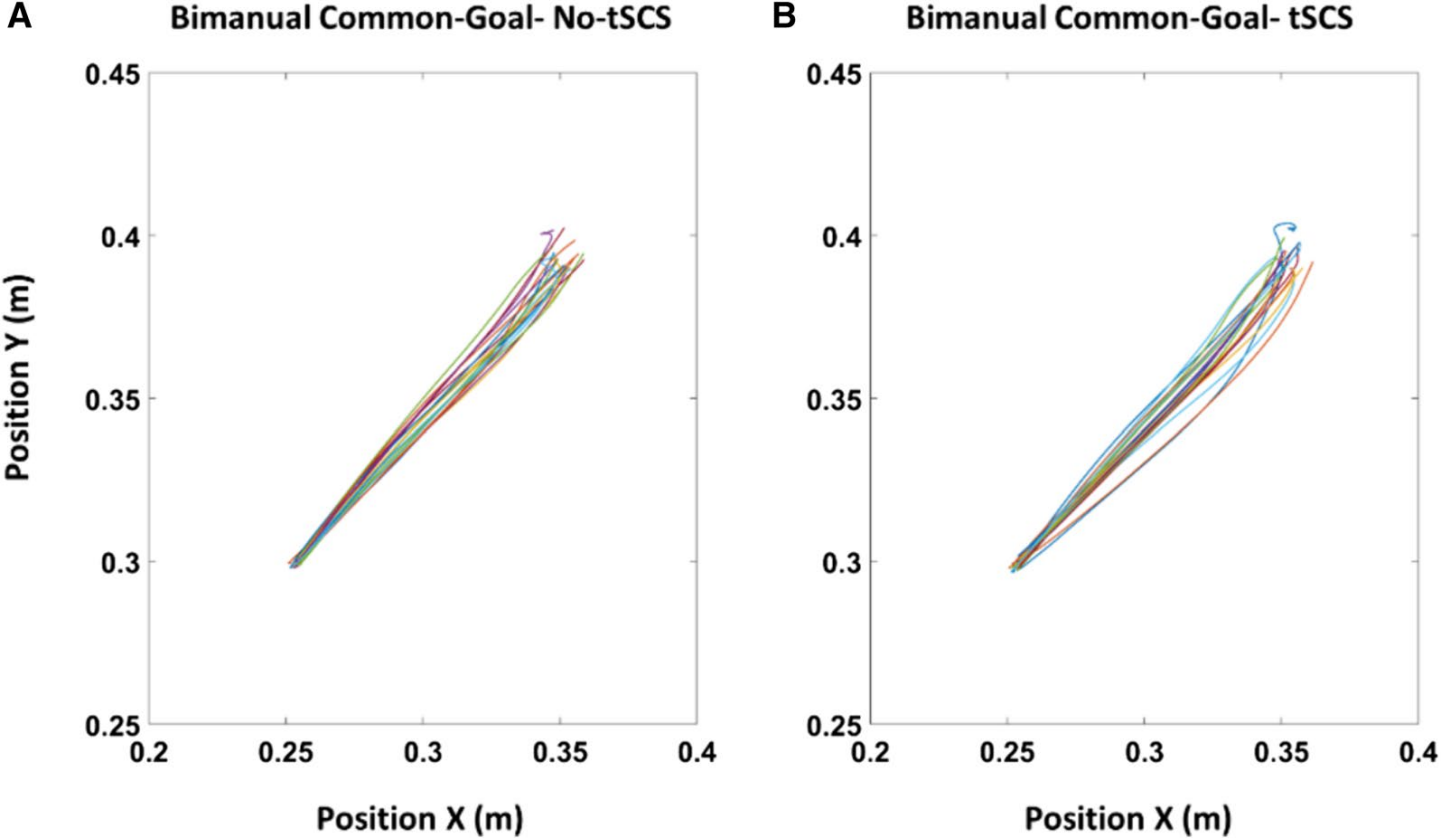
Right arm raw movement traces during the execution of bimanual common-goal task in X–Y coordinates **A** when tSCS was off, and **B** when tSCS was applied to the cervical spinal cord from a representative participant. Each color is a single reaching movement, and each plot illustrates an overall of 20 repetitions per task

**Fig. 4:**
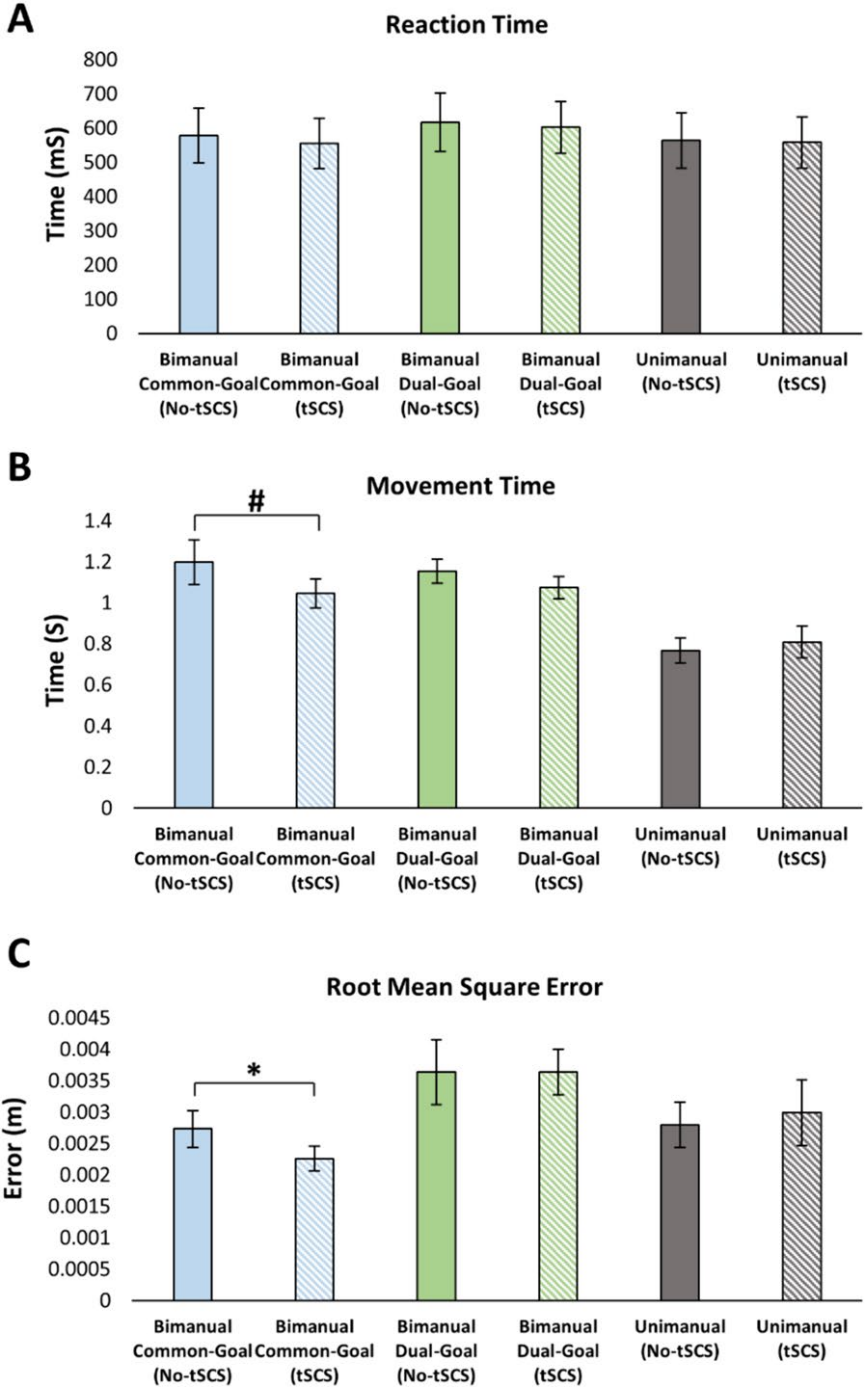
Relationship between movement kinematics and stimulation. Values are Mean ± SE. Cervical tSCS did not significantly alter **A** reaction time, **B** movement time, and **C** movement error relative to the no tSCS condition. (*P < 0.05; ^#^*P* < 0.1)

Figure 3 illustrates right arm traces of a reaching movement with and without tSCS from a representative participant. In partial agreement with our hypothesis, tSCS applied to the cervical spinal cord significantly decreased movement error (RMSE) relative to when tSCS was off during the bimanual common-goal task (*p* = 0.010) (Fig. 4C). In addition, MT was faster with tSCS during the bimanual common-goal movement and approached significance (*p* = 0.072) (Fig. 4B). In a partial contradiction to our hypothesis, cervical tSCS had no effect on other movement kinematic measures. Cervical tSCS had no effect on RT for any of the tasks [common-goal (*p* = 0.217), dual-goal: (*p* = 0.458), unimanual: (*p* = 0.702)] (Fig. 4A), and tSCS had no significant effect on MT for the bimanual dual-goal or unimanual movements [dual-goal: (*p* = 0.238), unimanual: (*p* = 0.457)] (Fig. 4B). Movement error (RMSE) was not affected by tSCS for the bimanual dual-goal and unimanual movements [dual-goal: (*p* = 0.992), unimanual: (*p* = 0.468)] (Fig. 4C).

### Sensorimotor spectral power and coherence after artifact removal

**Fig. 5:**
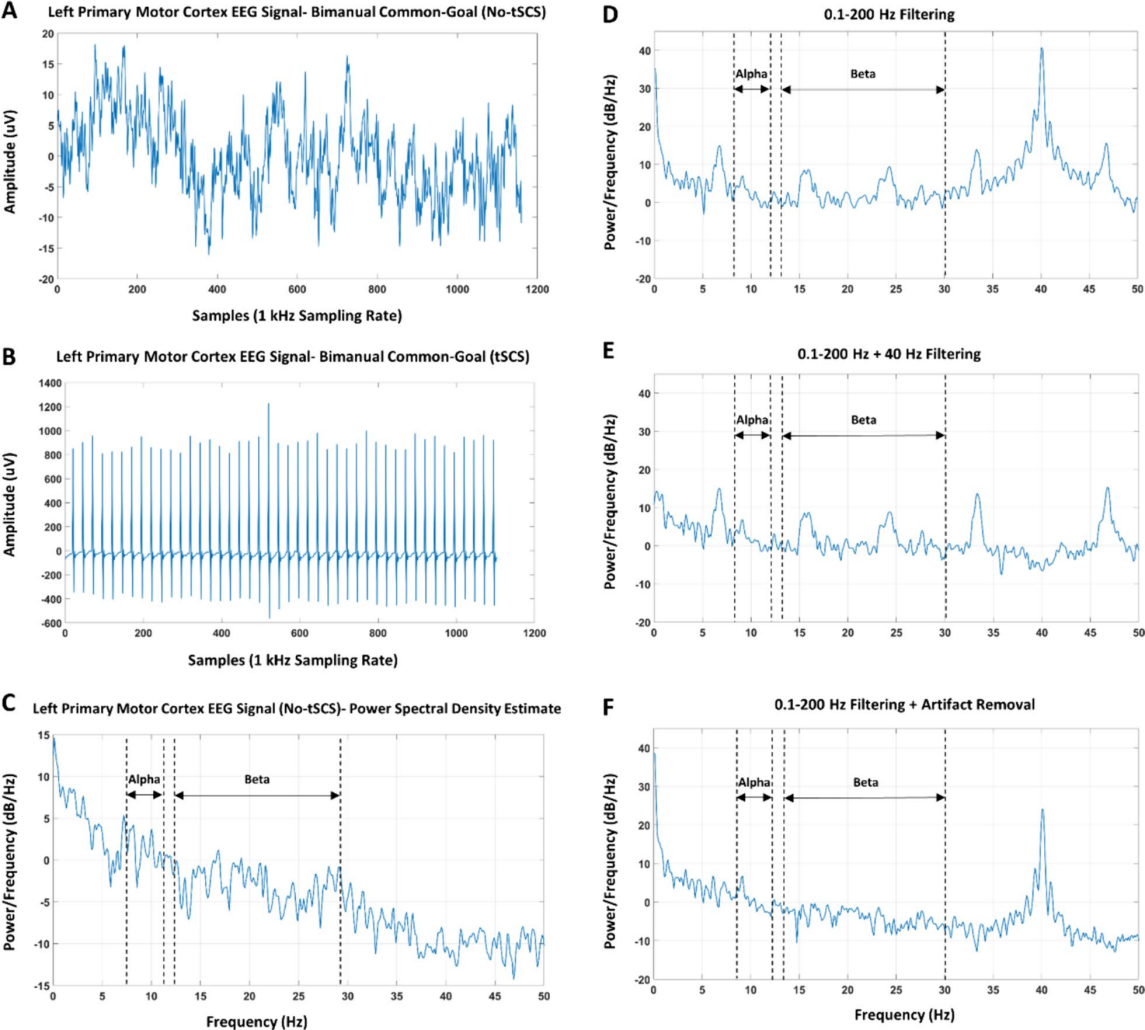
Raw EEG signal and power spectral density with different filtering approaches and artifact removal from a representative participant. Single trial EEG signal recorded from the left primary motor cortex during the execution of bimanual common-goal task (**A**) without tSCS, (**B**) in the presence of tSCS. (**C**) Power spectrum of the concatenated EEG signal (a single trial of it is shown in **A**) without tSCS. **D** Representative welch power spectral density estimate of the EEG signal recorded from left primary motor cortex with only basic band-pass filtering of 0.1–200 Hz. **E** Power spectral density of the same EEG signal with the addition of 40 Hz notch filtering. **F** Power spectral density of the artifact-free EEG signal. A general reduction of spectral power is observed in both the alpha and beta bands when the transient high-amplitude peaks are eliminated

Figure 5A and B depict examples of single trial raw EEG signal in the absence and presence of tSCS, respectively. An example of the welch power spectral density estimate before and after artifact removal from the C3 electrode during the execution of the common-goal task from one representative participant is provided in Fig. 5D, F. These figures show the effectiveness of the artifact removal technique used in correcting the transient abnormal peaks in the EEG power spectrum.

**Fig. 6:**
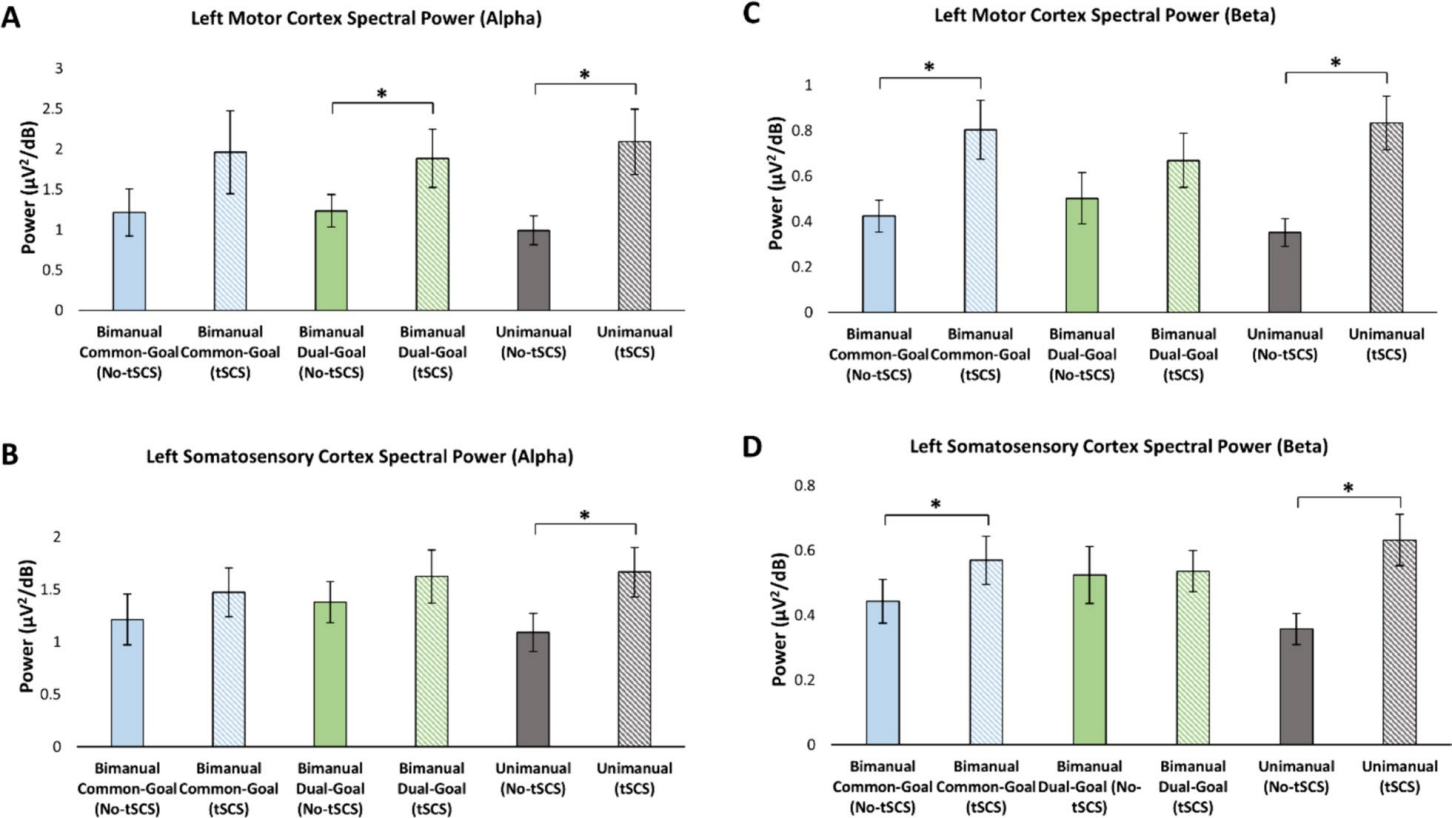
Alpha and beta band spectral power analysis. Alpha (**A**, **B**) and beta band (**C**, **D**) spectral power during the execution of the three movement tasks with and without tSCS. An augmentation in spectral power is seen in the alpha band during both unimanual and dual-goal movements in the presence of tSCS. Additionally, elevated spectral power is observed in the beta band for unimanual movement, as well as in the beta band during common-goal movement

In the alpha band, cervical tSCS yielded a significant increase of spectral power over the C3 electrode during dual-goal bimanual (*p* = 0.033) and unimanual movements (*p* = 0.005), but not during common-goal bimanual movement (*p* = 0.144) (Fig. 6A). We also observed a significant increase in spectral power when stimulation was present in the alpha band over the CP3 electrode during the unimanual task, but not during the common-goal and dual-goal bimanual movements [common-goal (*p* = 0.376), dual-goal: (*p* = 0.207), unimanual: (*p* = 0.005)] (Fig. 6B). Significant increases in spectral power in the beta band in the presence of tSCS (relative to No-tSCS condition) was found over the C3 electrode during the execution of the common-goal bimanual (*p* = 0.001) and unimanual (*p* < 0.001) movements (Fig. 6C). Similarly, cervical tSCS led to an increase in the beta band spectral power over the CP3 electrode ([common-goal (*p* = 0.028), unimanual: (*p* = 0.001)] (Fig. 6D). There were no significant differences in C3 (*p* = 0.097) and CP3 (*p* = 0.837) beta band spectral power in the dual-goal task when tSCS was delivered compared to when it was absent.

**Fig. 7:**
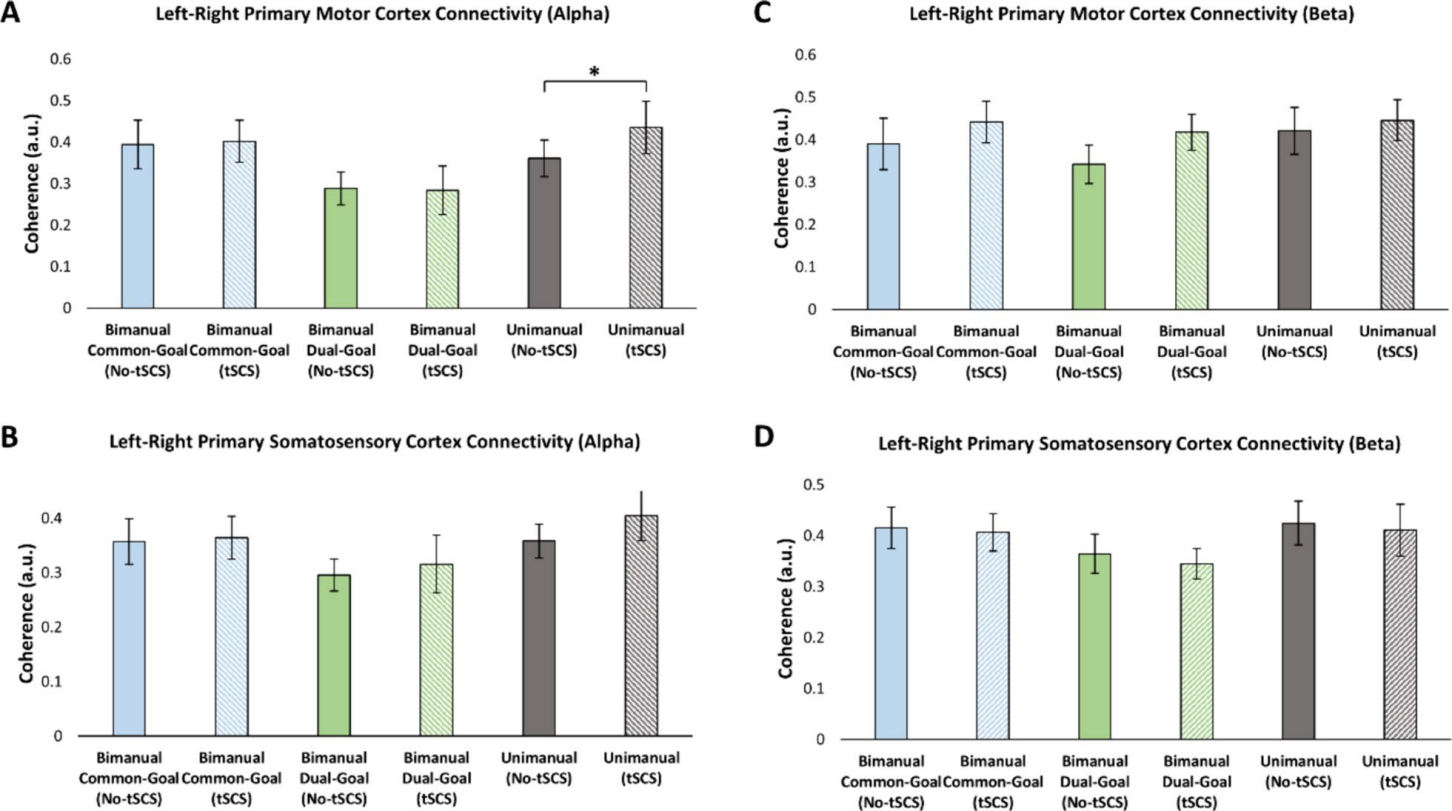
Interhemispheric connectivity with artifact removal applied between left and right (**A**, **C**) primary motor cortex, and (**B**, **D**) primary somatosensory cortex. The alterations in interhemispheric connectivity do not exhibit consistency across frequency bands and tasks. A general elevation of interhemispheric connectivity is evident in the alpha band. However, a reduction in interhemispheric coupling is observed in the beta band, specifically over the somatosensory cortex

In the alpha band, a significant increase in C3–C4 coherence was found during the unimanual task when tSCS was present (*p* = 0.043), but not during common-goal and dual-goal bimanual tasks [common-goal (*p* = 0.825), dual-goal: (*p* = 0.922)] (Fig. 7A). CP3–CP4 coherence in the alpha band was not affected by the application of cervical tSCS relative to when tSCS was not present for all movement tasks [common-goal (*p* = 0.812), dual-goal: (*p* = 0.629), unimanual: (*p* = 0.285)] (Fig. 7B). In the beta band, coherence between C3 and C4 electrodes was not significantly different between tSCS and No-tSCS conditions regardless of the task [common-goal (*p* = 0.225), dual-goal: (*p* = 0.149), unimanual: (*p* = 0.473)] (Fig. 7C). Similarly, no difference in the CP3–CP4 beta band coherence was found between tSCS and No-tSCS conditions [common-goal (*p* = 0.804), dual-goal: (*p* = 0.641), unimanual: (*p* = 0.725)] (Fig. 7D).

## Discussion

In the current study, we examined the modulation of the cortical mechanisms involved in unimanual and bimanual tasks in the presence of cervical tSCS. The choice of studying the M1 and S1 sensorimotor cortical regions was based on the pivotal role of these areas in modulating bimanual performance [39]. We found that beta cortical oscillations associated with left sensorimotor regions were significantly modulated by tSCS during the execution of both unimanual and bimanual common-goal movements, pointing to the increase in synchronous neural firing in M1 and S1 induced by tSCS. In the alpha band however, we observed ERS of sensorimotor cortical activity only during unimanual movement. Our finding demonstrated that there is no significant modulation of interhemispheric connectivity between left and right M1 and S1 when cervical tSCS was applied. Furthermore, our study revealed that cervical tSCS improved performance during the bimanual common-goal task as characterized by MT and RMSE, but had no effect on movement kinematics during the execution of bimanual dual-goal and unimanual tasks. To the best of our knowledge, this is the first study to investigate the neural correlates of three behaviorally distinct unimanual and bimanual tasks under the influence of cervical tSCS using EEG measures. Up until now, knowledge about the effect of tSCS on cortical networks underlying bimanual motor control as well as cortical neurophysiological mechanisms of tSCS was very limited.

A number of studies over the past few years demonstrated that tSCS may be effective in improving sensorimotor function after SCI [14, 23, 40]. These studies used metrics such as spinally-evoked potentials, MEPs [34], cervicomedullary evoked potentials (CMEP) [3], and H-reflexes [1, 37] to investigate the underlying mechanisms of this electrical stimulation neuromodulatory technique. Only one previous study provided information regarding the effect of tSCS on cortical oscillations captured by EEG. McGeady et al. reported that 10 min of cervical tSCS is not sufficient to produce significant modulation of sensorimotor brain rhythm, but this finding was not consistent among all participants and relied on the intensity of stimulation [32]. Participants who received the highest doses of stimulation had suppression of cortical activity (10% ERS), implying that stimulation intensity is a critical factor at cortical level. In line with this view, a crucial finding in our work was a significant suppression of sensorimotor cortical activity in some of the performed tasks. Nonetheless, there are two important differences between our procedure for electrode placement and determining stimulus intensity and the work by McGeady and colleagues. First, we placed two adhesive cathodic electrodes midline at C3–4 and C6–C7 [37], instead of single electrode at C5–6 as was the case in the McGeady et al. study [32]. Second, instead of subjectively setting the current intensity by asking the participants about their maximum tolerance level, we followed the procedure outlined in Benavides et al., in which stimulation intensity was determined based on the threshold that induces spinally-evoked potentials [3]. In addition, we used a stimulation frequency of 40 Hz instead of the 30Hz used in McGeady et al. [32].

This suppression of cortical activity is not surprising in this study. We previously reported unchanged MEPs in the presence of cervical tSCS with 10 kHz modulation [37]. However, a recent study determined that tSCS with 5 kHz carrier frequency facilitated the amplitude of CMEPs but did not modulate the amplitude of MEPs [3], and suggested that tSCS activates cortical inhibitory networks projecting to corticospinal neurons. Interestingly, the facilitation of MEPs in Benavides et al. only happened when the carrier frequency was removed from the stimulation waveform, suggesting that the carrier frequency contributed to the inhibitory mechanism [3]. This effect was further substantiated by an increase in the level of short-interval intracortical inhibition (SICI) only when tSCS was applied with 5 kHz carrier frequency [3]. It has been suggested that the modulation of SICI is mediated by intracortical GABA inhibitory networks [11]. Therefore, with the presence of 10 kHz carrier frequency in our study, it is rational to contemplate that a similar inhibitory intracortical mechanism is responsible for the suppression of motor and sensory cortical activity (i.e., ERS) found in this study.

Alternatively, the ERS may be a consequence of exposure to discomfort caused by stimulation [29, 38]. Participants in our study verbally reported a strong fluttering or vibration sensation at the cathodic sites. The amplitude of stimulation for each participant was also close to maximal tolerance; at this level the participants could not tolerate the stimulation for more than 3–4 min (the duration of the task). Maximal tolerance with tSCS applied laterally across the spinous process between lumbar L1 and L2 vertebrae was shown to be more than 50% lower than the stimulation level required to elicit spinally-evoked potentials [30]. The stimulation amplitude in our study was set similarly (i.e., at the level that induces spinally-evoked potentials), which would have caused experience of discomfort. Moreover, discomfort and painful sensations are associated with reduced ERD during movement [49]. Thus, discomfort experienced by the participants may have contributed to the suppressive effect on cortical activity observed in this study.

Parallels can be drawn from neuromuscular electrical stimulation (NMES) studies. Modulation of brain activation induced by NMES has been reported previously [45, 47]. For example, NMES of wrist extensor induced stimulation intensity dependent modulation of sensorimotor cortical activity, with above motor threshold intensities producing cortical facilitation and below motor threshold intensities causing cortical inhibition [24]. Importantly, motor threshold level in the NMES study is defined as the intensity that induces finger twitches, and at this level proprioceptive receptors as well as cutaneous mechanoreceptors are activated [4, 17]. With below motor threshold stimulation, however, only cutaneous mechanoreceptors are activated [24]. The procedure to determine the amplitude of stimulation ensured that all the participants in our study received stimulation at the level that elicits spinally-evoked potentials. At this level, posterior root afferents are recruited [2, 36]. Thus, we may conclude that tSCS through recruitment of posterior root afferents should produce the same facilitation of ERD observed in the NMES study; however, we found the opposite. This effect may be due to exposure to high-intensity stimulation and activation of intracortical inhibitory networks which could have interfered with the conduction of sensory information [3, 32].

We did not find significant modulation of interhemispheric connectivity in cortical sensorimotor regions. Our results suggest a trend towards increased beta band interhemispheric connectivity between left and right M1when tSCS was delivered relative to when tSCS was off across all movement conditions, but the opposite of this trend was seen between the left and right S1 (i.e., decrease of interhemispheric connectivity). No particular trend was observed in the alpha band interhemispheric connectivity results. A recent study suggested both a decrease (in areas associated with direct motor control) and an increase (in areas of motor planning) of functional connectivity in the presence of lumbar tSCS during tonic and rhythmic muscle contraction of the lower limbs [48]. However, this effect was only observed at the level of cortical sources, and was absent for the EEG electrode-based analysis [48]. Similarly, NMES has been shown to strengthen interhemispheric functional connectivity between cortical sensorimotor regions [18]. Modulation of interhemispheric inhibition may explain increased/decreased functional connectivity between sensorimotor regions [7, 18]. The current investigation is unable to identify specific neural pathways or regions responsible for changes in the level of interhemispheric connectivity. Future investigation is needed using measures such as fMRI-based functional connectivity and TMS-based IHI to further explore tSCS-induced modulation of interhemispheric connectivity. Moreover, as suggested by Steele et al. [48], connectivity analysis is more accurate when performed at cortical source levels as opposed to when sensor-based information is employed.

Stimulation applied to regions near EEG electrodes is considered a major source of artifacts in the data and complicates the interpretation of the results. To alleviate the effect of stimulation artifacts in the EEG recordings, two approaches including artifact removal and inter-stimulus data extraction have been suggested previously [28]. The limitation of these approaches is the exclusion of brain data in the analysis during stimulation. To overcome this challenge, a recent study suggested that EEG during tSCS “bares statistically similar characteristics to that of normal EEG” if the frequency band of interest does not overlap with stimulation frequency [32, 33]. Therefore, no artifact removal techniques were thought to be necessary, but notch filtering was recommended in the frequency domain. In this study, we followed the procedure outlined in [32], which only involves applying a band-pass filter between 0.1 and 200 Hz. Additionally, we suppressed the tSCS-induced contamination of EEG data in the time domain by replacing artifacts with an average of clean EEG signal. This additional step was necessary because we observed the signs of stimulation artifact spreading beyond its frequency to nearby frequency bands (i.e., alpha and beta) in the spectral power analysis. This was evident as an abnormal brief peak near 20Hz in Fig. 5D. However, we acknowledge that removing parts of the EEG signal (the artifacts) and replacing them with average values, effectively alters the original dynamics of the signal during those intervals. This means removing not just the artifact but also deprivation of results from genuine brain signals during the stimulation period. The artifact removal approach led to a substantial reduction in both alpha and beta band tSCS-induced sensorimotor ERS relative to when only band-pass filtering was applied. This reduction of ERS demonstrates that retaining the simulation artifact in the data comes at the cost of exaggerated ERS, and hence misinterpretation of the results. Additionally, when a period of an EEG signal is flattened, this introduces discontinuities in the signal, which can manifest as spectral leakage in the frequency domain. Spectral leakage can lead to power being spread across different frequencies, which might influence spectral power estimates, not just at 40 Hz but to a broad range of frequencies. This is also a valid concern even if we used total removal of artifacts instead of replacing them with an average. The effect of artifact removal affects the immediate nearby frequencies, but as we move farther away from 40 Hz, the effect becomes less pronounced. Therefore, the selection of 40 Hz for the stimulation frequency aimed to separate the stimulation frequency from the frequency bands of interest, such as the beta band. Finally, we applied a windowing function before spectral power computation to limit the spectral leakage.

In this study, pulse duration was set to 1ms but the stimulation artifact recorded by EEG persisted for approximately 7–11ms. In other words, ~ 28–45% of the EEG data were replaced with an average of the clean EEG when the artifact removal approach was used. Thus, these two artifact removal approaches create a trade-off between the possibility of inaccurate frequency domain results and data loss. Notably, the type of tSCS stimulator used affects the duration of the resulting artifact. Using two DS8R stimulators for the cathodes in this study resulted in a relatively narrower artifact in the EEG recordings because the stimulus pulses were delivered simultaneously by the two stimulators. Delivering the stimulus pulses sequentially as is the case with other tSCS stimulators would increase the duration of the artifact to nearly twice the duration seen in our data, rendering any analysis of EEG activity virtually impossible.

We speculate that there are two underlying reasons contributing to the discrepancy between our view of handling stimulation artifact and what was suggested in McGeady et al. [32, 33]. First, stimulation frequency was 30 Hz in the McGeady et al. study [32, 33] compared to 40 Hz in this study. This means that the inter-stimulus interval was wider in the previous study allowing for ~ 25% higher amount of clean and useful data for frequency-domain analyses. Second, delivering stimulation through two cervical electrodes may have caused the pronounced stimulation artifact in the time series EEG data, which led to having only ~ 55–72% clean EEG signals between successive stimulation pulses. It is perhaps the case that with lower stimulation frequencies, tSCS presents no threat to frequency domain analyses as suggested by McGeady et al. [33], but artifact removal is required at higher frequencies. Future research is necessary to explore whether EEG recordings are feasible with tSCS at different stimulation frequencies, especially to assess how artifacts affect spectral power in frequency bands of interest such as alpha, beta, and gamma, as well as interhemispheric connectivity. EEG activity contaminated with stimulation artifacts can provide misleading representations in the frequency domain [48]. Thus, we believe that artifact removal is necessary, at least for higher stimulation frequencies such as 40 Hz.

Importantly, our results suggest that tSCS improves MT and RMSE of bimanual common-goal movements in participants with no history of neural injury or disease, but is ineffective in improving bimanual dual-goal and unimanual tasks. It has been previously shown than tSCS primarily activates afferent fibers of the dorsal roots and dorsal horn of the spinal cord [2, 15]. Through monosynaptic and oligosynaptic connections from sensory afferents, spinal α-motoneurons are recruited [9, 20]. We speculate that there is an increase in the transmission of proprioceptive information that enhanced the performance of the bimanual common-goal task. Successful performance of common-goal reaching movements requires extensive coordination between the two arms and constant sharing of spatial location between the two arms [12]. A recent study [10] investigated the activation of proprioceptive fibers during cervical transcutaneous spinal cord stimulation. This computational study suggests preferential activation of both Aα and Aβ fibers compared to α-motor fibers. This finding shows the contribution of both proprioceptive and cutaneous input to tSCS-evoked potentials. While this does not directly translate to enhanced proprioceptive function, we speculate that preferential activation of proprioceptive fibers may have contributed to better performance of the bimanual common-goal task which relies on proprioception more than the other two tasks. Therefore, it is plausible that the increased proprioceptive input during cervical tSCS contributed to improved kinematics particularly during bimanual common-goal movements.

Although improvements in unimanual hand and arm function were previously reported with tSCS after SCI [23], the reason improvements in kinematic performance were not seen during the unimanual and dual-goal tasks in this study is likely because the participants were neurologically intact. Nonetheless, our current findings critically highlight the potential of tSCS in promoting recovery of bimanual movements after neurological conditions. If tSCS is capable of improving movement accuracy and movement time in participants with no history of neural injuries/diseases, it is possible that kinematic outcomes can be improved for participants with SCI or stroke. We posit that this behavioral improvement can be achieved through hybrid rehabilitation training that consists of bimanual coordination tasks and tSCS. Moreover, our findings highlight the importance of comprehensively and accurately assessing bimanual impairments and quantifying bimanual performance after SCI/stroke. Stroke survivors exhibit varying performance levels when engaged in different bimanual movements [26]. Elucidating what aspect of bimanual movements is primarily targeted by tSCS in participants with neurological conditions is a question for future studies.

If the kinematic performance of common-goal movements is improved with tSCS in people with SCI or stroke, analogous to what the present study found, our results could serve to inform the optimal bimanual rehabilitation training design. Our task design offers a precise and sensitive measure for kinematic analyses of arm function before and after rehabilitation training. At this time, it is difficult to make a conclusion about the link between cortical synchronization and potential behavioral improvement caused by tSCS when tested in a clinical population. The key is to track the changes in the level of cortical ERS (or ERD) during the course of a tSCS-based bimanual rehabilitation training paradigm and correlate it with behavioral improvements. Our study suggests sensorimotor cortical inhibition when tSCS is applied with a modulated waveform (i.e., 10 kHz waveform). A previous study suggested stronger corticospinal excitability after the application of tSCS for 20 min only when the kHz modulation was removed [3]. Another study found no significant changes in corticospinal excitability with stimulation at 40% of the posterior root reflex (PRR) threshold, but found modulation of MEPs at 60% and 80% threshold [31]. Taken together, our present work and these two previous studies demonstrate that cervical tSCS using a 10 kHz carrier frequency influences cortical activation and excitability. While it is not possible to draw definitive conclusions from the results of this study and previous work at this time, it seems that tSCS, when administered at a sufficient intensity level tailored to the specific stimulation paradigm, can increase corticospinal excitability. Moreover, it may be the case that a non-modulated tSCS waveform that does not cause cortical inhibition and leads to stronger corticospinal excitability is more beneficial for improving upper limb function, especially performance of bimanual common-goal movement, after neural injury.

## Study limitations

This study has three primary limitations. First, while we tested the effects of tSCS on movement performance and cortical activity during stimulation, short- or long-term effects on movement performance in the absence of stimulation were not evaluated. It is important to determine whether the stimulation in this study exerts its influence only during the administration period or it provides short and/or long-lasting effects after it is switched off. Moreover, the tasks performed with stimulation turned off could potentially be influenced by a carry-over effect from previous tasks where stimulation was active. Despite the random presentation of tasks to participants, the brief intervals between them might have contributed to some degree of carry-over effects. This potential carry-over effect could lead to changes in the outcomes of tasks performed without stimulation, resulting in only minimal differences between the conditions.

Second, measures such as IHI or SICI were not tested; such measures can provide valuable knowledge regarding intracortical inhibitory and excitatory interactions and circuits. Since the underlying neurophysiological mechanisms of IHI and SICI are known [42], these methods directly inform us of intra-hemispheric and interhemispheric connections. They can also serve a comparative measure to corroborate the results obtained by EEG connectivity analysis.

Third, the study was conducted in neurologically intact participants; future studies in persons with neural injury or disease such as SCI, stroke or multiple sclerosis would unravel the effects of these neurological conditions on cortical activity and kinematics of bimanual arm movements and the potential benefits of tSCS. Multiple studies involving clinical populations reported marked improvement of function after tSCS-based rehabilitation [3, 15, 23, 44], with one study noting long-term benefits [23]. A ceiling effect imposes a limit on the recruitment of additional fibers in neurologically intact participants where the nervous system is being utilized to its fullest extent [32]. When the nervous system is fully functional due to the absence of neural injury or disease, modulation of outcome electrophysiological and kinematic measures may not be overtly present with tSCS. Thus, assuming the effectiveness of tSCS, it is reasonable to anticipate observing the most pronounced effects in clinical populations.

Future work should also investigate the effects of tSCS at different frequencies (such as 30 Hz) allowing for wider inter-stimulus intervals and using EEG source identification methods to accurately localize cortical regions of interest along with their corresponding activation level and connectivity to other sources. At 40 Hz, the stimulation artifacts crept into the spectral power density and contaminated the frequency bands of interest. The 1ms tSCS pulses in the time domain resulted in 7 to 11 ms artifacts in the EEG recording. Therefore, as opposed to what was suggested in McGeady et al. [33], stimulation is a threat to both time and frequency domain analyses and an artifact cleaning/removal measure should be implemented. Finally, the age group of 19–36 was primarily due to our recruitment strategy from the University of Alberta students and staff who were available to us during the COVID-19 pandemic. We believe that while this might limit the generalizability of our findings, the results we observed are applicable to broader age groups. Nonetheless, motor and cognitive decline associated with aging can affect both kinematic and electrophysiological results.

## Data Availability

The datasets used and/or analyzed for this study will be available on open data commons platforms such as the Open Data Commons for Spinal Cord Injury (https://odc-sci.org/). Other data will be available from the corresponding author upon reasonable request.
